# NMJ-morph reveals principal components of synaptic morphology influencing structure–function relationships at the neuromuscular junction

**DOI:** 10.1098/rsob.160240

**Published:** 2016-12-07

**Authors:** Ross A. Jones, Caitlan D. Reich, Kosala N. Dissanayake, Fanney Kristmundsdottir, Gordon S. Findlater, Richard R. Ribchester, Martin W. Simmen, Thomas H. Gillingwater

**Affiliations:** 1 Euan MacDonald Centre for Motor Neurone Disease Research, University of Edinburgh, Old Medical School, Teviot Place, Edinburgh EH8 9XD, UK; 2 Centre for Integrative Physiology, University of Edinburgh, Old Medical School, Teviot Place, Edinburgh EH8 9XD, UK; 3 Anatomy, Edinburgh Medical School: Biomedical Sciences, University of Edinburgh, Old Medical School, Teviot Place, Edinburgh EH8 9XD, UK

**Keywords:** synapse, neuromuscular junction, morphology, statistical modelling, NMJ-morph

## Abstract

The ability to form synapses is one of the fundamental properties required by the mammalian nervous system to generate network connectivity. Structural and functional diversity among synaptic populations is a key hallmark of network diversity, and yet we know comparatively little about the morphological principles that govern variability in the size, shape and strength of synapses. Using the mouse neuromuscular junction (NMJ) as an experimentally accessible model synapse, we report on the development of a robust, standardized methodology to facilitate comparative morphometric analysis of synapses (‘NMJ-morph’). We used NMJ-morph to generate baseline morphological reference data for 21 separate pre- and post-synaptic variables from 2160 individual NMJs belonging to nine anatomically distinct populations of synapses, revealing systematic differences in NMJ morphology between defined synaptic populations. Principal components analysis revealed that overall NMJ size and the degree of synaptic fragmentation, alongside pre-synaptic axon diameter, were the most critical parameters in defining synaptic morphology. ‘Average’ synaptic morphology was remarkably conserved between comparable synapses from the left and right sides of the body. Systematic differences in synaptic morphology predicted corresponding differences in synaptic function that were supported by physiological recordings, confirming the robust relationship between synaptic size and strength.

## 1. Background

The establishment and maintenance of network connectivity through the formation of appropriate synaptic connections is a fundamental determinant of function in the mammalian nervous system [1,2]. Any attempt to understand the complex structure and function of the nervous system therefore requires precise and detailed mapping of synaptic connectivity (‘connectomics’) in order to reveal essential characteristics of neural circuits that would otherwise be inaccessible [3,4]. The critical role played by synapses in maintaining network function is further illustrated by observing the catastrophic consequences of synaptic dysfunction and loss in neurodegenerative conditions such as Alzheimer's disease, Parkinson's disease and motor neuron disease [5–7].

Structural and functional diversity among synaptic populations is one of the key hallmarks of network diversity. However, we know comparatively little about the over-arching morphological principles that govern variability in the size, shape and strength of synapses. For example, although the influence of individual genetic and environmental factors on synapses has been explored in many studies [8–10], the extent to which such influences combine to control synaptic morphology remains unclear, and the hierarchical nature of these influencing factors remains to be established. A better understanding of these principles is therefore required in order to fully appreciate the identity and role of network connectivity within the nervous system in health and disease.

Given the complexity of synaptic connectivity that exists within the mammalian central nervous system (CNS) [2], it is difficult to obtain accurate three-dimensional morphological measurements from a sufficient number of identified synapses to undertake comparative morphometric studies. By contrast, the neuromuscular junction (NMJ), a synapse formed between a lower motor neuron in the peripheral nervous system and its target skeletal muscle fibre, offers an experimentally accessible model, well suited to studies of synaptic morphology and connectivity, and from which insights applicable to synaptic biology throughout the nervous system have been previously obtained [11]. In addition, just like synapses in the CNS, the NMJ represents a site of primary pathology in many neurological conditions, including myasthenia gravis and related disorders [12,13], congenital myasthenic syndromes [14], and motor neuron diseases such as amyotrophic lateral sclerosis (ALS) and spinal muscular atrophy (SMA) [15].

The basic cellular structure of synapses at NMJs is well established [16], and its structure is evidently adapted to allow robust and reliable neurotransmission with a high ‘safety factor’ [17]. Historically, the earliest morphological descriptions of the NMJ used gold and silver preparations to demonstrate plate-like (en plaque) [18] and grape-like (en grappe) [19] nerve terminals in various vertebrate species. With the advent of cholinesterase staining in the 1950s (developed by Koelle & Friedenwald [20]), visualization of both pre- and post-synaptic components of the NMJ became possible. Subsequent studies of the innervation of muscle in various mammalian species took advantage of these combined techniques [21], leading to a wide variety of qualitative methods for categorizing nerve terminals [22] and motor endplates [23]. Scanning electron microscopy confirmed that this cellular arrangement is conserved across NMJs in species as diverse as the frog, zebra finch and Chinese hamster, albeit with substantive differences in overall morphology [24]. More recently, the basic cellular triad of motor nerve terminal, skeletal muscle fibre and terminal Schwann cell(s) has been expanded to include a putative fourth cell type: ‘kranocytes’ [25] (so-named because of their anatomical relationship to the NMJ, where they are found ‘capping’ the aforementioned structures).

With the advent of immunofluorescent techniques and confocal microscopy, coupled with the wide availability of computer software, the possibilities for undertaking robust, quantitative morphometric analysis of the NMJ have advanced significantly. However, as yet, no standard methodology for comparative morphometric analysis of the NMJ has emerged, obstructing attempts to discover or test hypotheses concerning fundamental morphological principles that may regulate or influence synaptic structure–function relationships. In this study, we report on the development and testing of a new, ImageJ-based platform to allow standardized morphometric analysis of NMJs (‘NMJ-morph’). By applying NMJ-morph to mouse NMJs, we have generated baseline morphological reference data for 21 separate pre- and post-synaptic variables from 2160 individual NMJs belonging to nine anatomically distinct populations of synapses. Despite the marked heterogeneity of NMJs, we show how use of NMJ-morph, in conjunction with statistical analysis, reveals systematic differences in NMJ morphology between defined synaptic populations in skeletal muscle, and that the sensitivity of the approach is sufficient to distinguish between subtly different populations of NMJs in accordance with predicted physiological recordings.

## 2. Material and methods

### 2.1. Muscle dissection and neuromuscular junction immunohistochemistry

Twelve mice (six male, six female) were selected at random from a single litter of CD1 wild-type mice at six weeks of age. Following euthanasia by overdose of inhaled isoflurane, each mouse was weighed, and the chosen muscles from each side were dissected out, within 30 min post-mortem. Muscles were immediately fixed in 4% paraformaldehyde for 30 min, then washed in 1% phosphate buffered saline (PBS). All remaining connective tissue was then removed. Cranial and lumbrical muscles intended for whole-mount were immediately prepared for immunohistochemistry (see below). The triceps and quadriceps were cryoprotected by immersion in 30% sucrose overnight; 100 µm sections were then obtained on a Thermo Scientific Microm HM 450/KS 34 freezing microtome.

Neuromuscular junctions were immunohistochemically labelled using a standard laboratory protocol for visualizing pre-synaptic 2H3/SV2 and post-synaptic AChRs [26]. Muscle preparations were placed in the following sequence of solutions (made up in 1% PBS unless otherwise specified; antibodies and their concentrations are listed below): α-bungarotoxin (BTX) for 30 min to label post-synaptic AChRs; 4% Triton X for 90 min; a blocking solution of 4% bovine serum albumin (BSA) and 2% Triton X for 30 min; the primary antibodies (made up in blocking solution) for 72 h at 4°C; 1% PBS for 80 min; 4% BSA for 30 min; the secondary antibodies (made up in 1% PBS) for 150 min; 1% PBS for 80 min. Finally, muscles preparations were mounted on glass slides in Mowiol, and stored at −20°C. At all stages, samples were protected from excessive light exposure prior to imaging.

### 2.2. Antibodies

*Primary antibodies:* 1 : 50 mouse anti-SV2 (synaptic vesicles) IgG, 1 : 50 mouse anti-2H3 (neurofilament 165) IgG (both from Developmental Studies Hybridoma Bank). *Secondary antibodies:* 1 : 100 Cy3 AffiniPure donkey anti-mouse IgG (Jackson ImmunoResearch Labs). *BTX*: 1 : 500 *α*-bungarotoxin CF488A (Biotium).

### 2.3. Confocal microscopy

Images were acquired on a Zeiss LSM 710 confocal microscope. Confocal settings were optimized to achieve the best compromise between image quality and acquisition rate: 8 bit depth, 512 × 512 frame size, ×63 magnification, ×2 zoom and 1 µm z-stack interval, with sequential image acquisition to minimize bleed through (red channel—543 nm excitation, 565–615 nm collection; green channel—488 nm excitation, 500–550 nm collection). All image analysis was performed on maximum intensity projections of the z-stacks, using ImageJ software and the BinaryConnectivity plugin.

### 2.4. NMJ-morph

The workflow developed here (‘NMJ-morph’) is platform independent, and can be used on Windows or Mac operating systems. The latest version of ImageJ and the BinaryConnectivity plugin are freely available in the public domain, and can be downloaded at http://imagej.nih.gov/ij and http://www.mecourse.com/landinig/software/software.html (under the section ‘Morphological Operators for ImageJ’). The online supporting material includes a comprehensive NMJ-morph User Guide (which incorporates a step-by-step guide to performing each measurement). This file along with spreadsheet templates and a sample image bank for use in training and standardization are freely available for download from http://dx.doi.org/10.7488/ds/1490.

NMJ-morph is designed to facilitate quantitative analysis of both pre- and post-synaptic structures at the NMJ, based on a standardized and repeatable workflow suitable for use on confocal z-stack projections of individual NMJs. If confocal microscopy is either not possible (e.g. in simultaneous recording of structure–function from the same NMJs) or unavailable to some workers, a similar standardized approach using wide field fluorescence microscopy can be utilized for NMJ-morph analysis (data not shown), but confocal z-stack projections are recommended for optimal results. Table 1 details the complete set of 21 morphological variables that are included in the NMJ-morph workflow. In our hands, trained users were able to obtain comprehensive morphometric data from more than 30 NMJs per hour.

**Table 1.** Morphological variables and baseline data**.** A total of 21 morphological variables are included in the NMJ-morph protocol. Maximum and minimum values for each NMJ variable correspond to the mean values per muscle (240 NMJs) for the data shown in figure 4 and electronic supplementary material, figure S4. The fold difference between maximum and minimum values was significant for all variables except the average number of axon inputs (****p < 0.0001, ***p < 0.001, **p < 0.01, *p < 0.05).

	maximum	minimum	fold difference
core variables			
*pre-synaptic*			
(1) nerve terminal area (µm^2^)	287.1	159.0	1.81****
(2) nerve terminal perimeter (µm)	409.2	192.0	2.13****
(3) number terminal branches	47	22	2.14**
(4) number branch points	32	19	1.68**
(5) total length branches (µm)	191.0	103.1	1.85****
* post-synaptic*			
(6) AChR area (µm^2^)	402.3	168.4	2.39****
(7) AChR perimeter (µm)	400.4	199.1	2.01****
(8) endplate area (µm^2^)	834.0	273.6	3.05****
(9) endplate perimeter (µm)	134.2	71.2	1.88****
(10) endplate diameter (µm)	43.0	24.6	1.75****
(11) number AChR clusters	7.3	3.4	2.15**
derived variables			
* pre-synaptic*			
(12) average length branches (µm)	6.1	4.5	1.35**
(13) complexity	5.28	4.52	1.17**
*post-synaptic*			
(14) average area AChR clusters (µm^2^)	185.0	58.3	3.17***
(15) fragmentation	0.78	0.53	1.47**
(16) compactness (%)	70.3	46.4	1.52**
(17) overlap (%)	71.4	57.9	1.23*
(18) area of synaptic contact (µm^2^)	266.0	115.2	2.31****
associated nerve and muscle variables			
(19) axon diameter (µm)	3.3	2.3	1.43***
(20) muscle fibre diameter (µm)	57.1	17.8	3.21****
(21) number of axonal inputs	1	1	1.00

**Figure 1.**
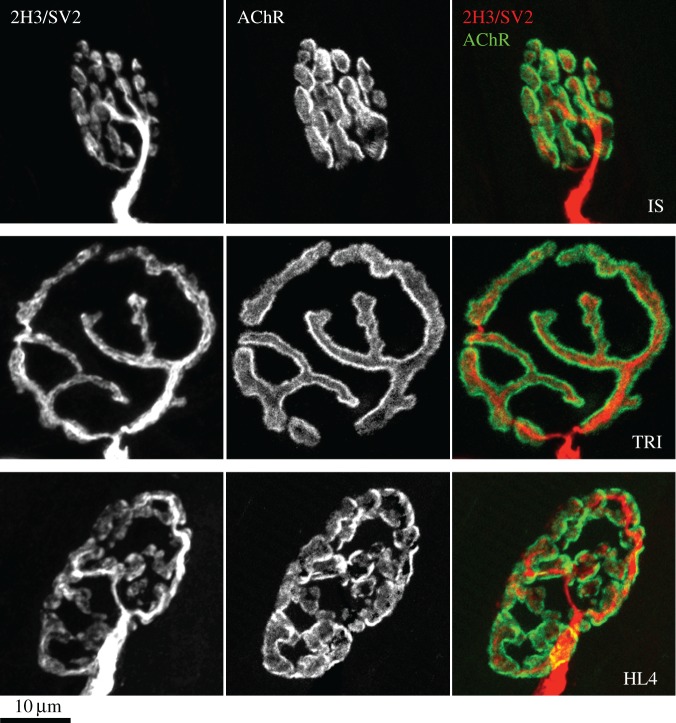
Morphological heterogeneity of mouse NMJs. Example NMJs from three body regions: head and neck (interscutularis muscle, IS), forelimb (triceps brachii, TRI) and hindlimb (4th deep lumbrical, HL4). The NMJs display marked differences in size and overall morphology, with no obvious relationship to body region. Scale bar = 10 µm.

Confocal micrographs of immunolabelled mouse NMJs were acquired from whole-mount muscle preparations, or 100 µm sections (for larger muscles), using standard image capture approaches (see above). For accurate analysis, each image captured a single en-face NMJ with a short length of its terminal axon in the centre of the field of view (figure 1). NMJs that were partially oblique to the field of view were only included if the oblique portion constituted less than approx. 10% of the total area.

Raw confocal images were then converted into binary counterparts. To set the threshold, two copies of the maximum intensity projection were opened simultaneously in ImageJ; the first was used as a reference, while selecting the threshold for the second. Several different thresholding methods are built in to ImageJ; in our experience, the ‘*Huang*’ method [27] provided the most accurate binary representation of the original image in 79% of NMJs (in an initial test series of 600 NMJs analysed by 2 independent investigators—see Results and electronic supplementary material, figure S1). For the remaining NMJs, the ‘*Huang*’ method did not provide accurate binary images; in these instances, the threshold was either set manually (for 18% of NMJs), or in the smallest number of cases (3%), we found the ‘*Yen*’ method [28] to be the next most useful automated threshold.

To ensure the very highest degree of reproducibility between datasets (or for those users who have less experience of NMJ-morph), we recommend that any images that cannot be accurately thresholded using the ‘*Huang*’ method should be discarded from the analysis at the outset. However, for those more familiar with NMJ-morph, the yield of the dataset can be increased by utilizing either manual thresholding or the ‘*Yen*’ method (as above) without compromising the quality of the data; in the initial test series of 600 NMJs, we found that these small, user-dependent differences in thresholding did not significantly influence the resulting analyses or reproducibility of data (see figure 3 and Results).

In situations where the signal-to-noise ratio of the images is lower than those used in this study (e.g. in pathological NMJs, during development, or in situations where NMJ structure and function are being studied simultaneously, and where intense labelling may not be consistent with normal function), it is likely that more reliance will be placed on manual thresholding, and greater variability between independent investigators will be inevitable. These more difficult circumstances also highlight the importance of thresholding one copy of an image with reference to the original, to ensure that the binary counterpart is an accurate representation of the original image.

One final point that we wish to highlight in relation to the use of NMJ-morph concerns the *absolute* accuracy of the measurements obtained. While the outlined workflow is both robust and repeatable *because* it uses thresholded, binary images (be they automatic or manually selected), the most accurate *absolute* measurement of individual variables (e.g. AChR cluster area) is only likely to be attained by manual measurements performed on the original, *unthresholded*, confocal images, whereby an individual can make subtle decisions about the precise boundaries of each NMJ. Inevitably, this is a vastly more time-consuming approach compared with the one outlined, and also subject to the inter-observer variability that we have sought to reduce, but for those groups dealing with smaller datasets, this may be the preferred option if time permits.

**Figure 2.**
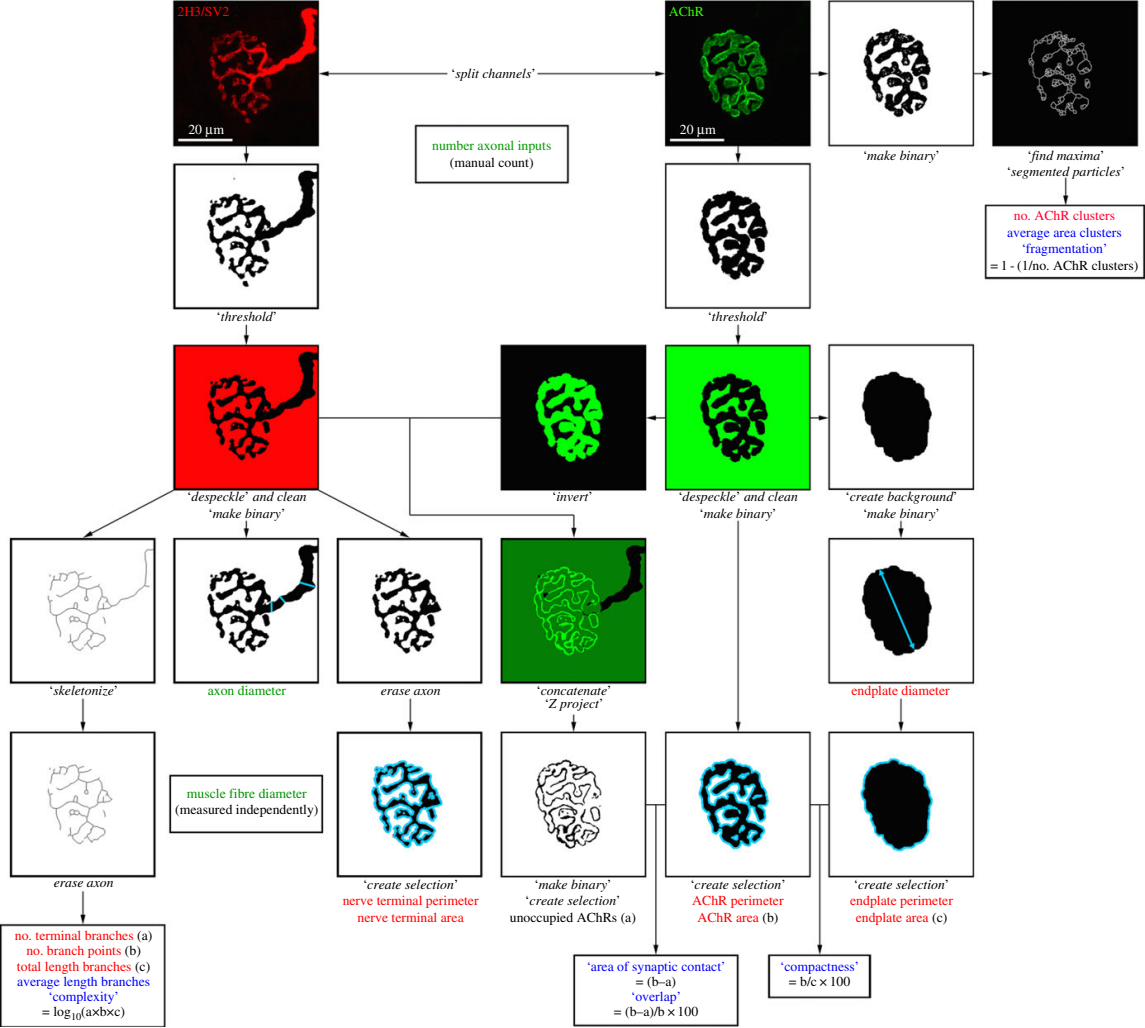
Overview of the NMJ-morph platform. Flowchart demonstrating the sequence of analyses for each NMJ. The workflow comprises 21 morphological variables: the 11 ‘core variables’ are shown in red typeface, the 7 ‘derived variables’ in blue and the 3 ‘associated nerve and muscle variables’ in green. Specific operations within NMJ-morph are shown in italics.

In total, we incorporated 21 individual morphological variables into the NMJ-morph platform, divided into ‘core variables’, ‘derived variables’ and ‘associated nerve and muscle variables’ (figure 2 and table 1). Basic, self-explanatory dimensions such as area and perimeter were measured using standard ImageJ functions (figure 2) (see online NMJ-morph User Guide for step-by-step instructions, available at: http://dx.doi.org/10.7488/ds/1490).

With regard to the more complex branching pattern of nerve terminals, previous studies have often used complex terminology to define branches (e.g. proximal and distal segments [29], orders of branching [30]), none of which lend themselves to easy or widespread use. We avoided such ambiguity in defining branches by using the ‘skeletonize’ function in ImageJ to produce a one-pixel-thick ‘skeleton’ of the nerve terminals. The number of terminal branches [31] and number of branch points were then calculated automatically using an ImageJ plugin: BinaryConnectivity [32] (electronic supplementary material, figure S2). This method was first described in relation to the branching pattern of the endplate [33], but has not previously been applied to the analysis of pre-synaptic nerve terminals.

The ‘complexity’ index was developed to combine the individual branch variables in a single pre-synaptic index (similar to the post-synaptic ‘compactness’ and ‘fragmentation’ indices; see below; figure 2). Previous methods for this type of analysis have varied from those using relatively complex nomenclature (e.g. average distance between points of origin of secondary branches on the primary branch [30]) to more straightforward methods (e.g. length of branches × number of branch points/100 [29]). We used the following formula with a logarithmic derivation to generate a more convenient numerical index:
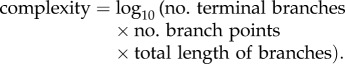


The basic dimensions of the post-synaptic motor endplate were again measured using standard ImageJ functions (figure 2; see online NMJ-morph User Guide for step-by-step instructions). More complex morphological variables of the post-synaptic motor endplate were established using several ‘derived’ variables (figure 2 and table 1). The ‘compactness’ of AChRs at the endplate was defined as follows:
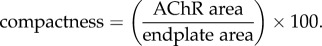


This index has been termed ‘dispersion’ in previous studies [33,34], but we favour the term ‘compactness’ as a more accurate descriptor of a quantity of receptors within a given area. Furthermore, it helps to draw a clear distinction between this variable and ‘fragmentation’ (see below), which describes a different physical characteristic of the endplate.

NMJ-morph was also used to quantify the number of discrete AChR clusters comprising the motor endplate. Although previous studies have reported simple counting of AChR receptor clusters [35], our experience suggested that this could be difficult in practice, as clusters lying in close proximity often appeared as one. We overcame this problem by utilizing the ‘segmented particles’ function in ImageJ, which resolved the endplate into completely discrete clusters that could be easily counted (electronic supplementary material, figure S3). This function is a type of ‘watershed segmentation’ for greyscale images that provides a means of automatically separating particles that touch [36]. Additionally, a ‘fragmentation’ index was calculated, whereby a solid plaque-like endplate has an index of zero (0), and a highly fragmented endplate has an index that tends towards a numerical value of one (1):
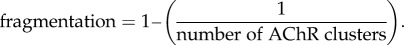


The remaining morphological variables measured using NMJ-morph (‘overlap’ and ‘area of synaptic contact’) provide details concerning the morphological ‘matching’ of the pre- and post-synaptic apparatus. ‘Overlap’ describes the degree of congruence between the pre- and post-synaptic elements of the NMJ—the extent to which the nerve terminal overlaps the AChR clusters. Partially occupied or vacant endplates are a hallmark of dying back pathology (e.g. ALS and SMA) and Wallerian degeneration [15], and previous studies have assigned NMJs on inspection to various categories, ranging from vacant to fully occupied, in a semi-quantitative manner [37]. The derived index ‘overlap’ allowed us to calculate the exact ‘percentage occupancy of AChRs’, using a method first described by Prakash *et al*. [30]:
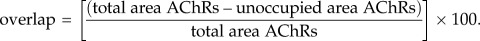


In a similar manner, we were able to ascribe a precise ‘area of synaptic contact’ for each NMJ as follows:
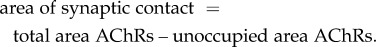


Measurements of muscle fibre diameter were obtained using an Olympus IX71 microscope/Hamamatsu C4742-95 camera set-up. Teased fibre preparations were imaged at ×20 magnification. Images were captured using Openlab Improvision software and measurement of muscle fibre diameter was performed manually in ImageJ. Forty individual fibres were measured per muscle (2160 muscle fibres in total for the complete dataset).

### 2.5. Electrophysiology

Intracellular recordings were made from within 100 µm of NMJs on muscle fibres in isolated IS and LAL nerve-muscle preparations bathed in HEPES-buffered mammalian physiological saline (MPS, containing 2 mM Ca^2+^ and 1 mM Mg^2+^) at room temperature (22–25°C), using glass microelectrodes filled with 4 M potassium acetate, resistances 20–40 MΩ, as described previously [38–40]. Muscle action potentials were blocked by pre-incubating muscles in µ-conotoxin GIIIB (Alomone, 2.5 µM in aerated MPS) for 20–30 min before transferring and pinning the preparations to a Sylgard-lined recording chamber mounted on the stage of an upright compound microscope (Zeiss Axioscop2 FS Plus) fitted with ×10–×40 water-dipping objectives. Nerve-evoked muscle responses were produced via a suction electrode connected to a Digitimer DS2 stimulator, applying timed pulses of 1–10 V amplitude and 0.1–0.2 ms duration. Spontaneous MEPPs and nerve-evoked EPPs were recorded with an Axoclamp 2B amplifier and digitized via a Digidata 1322A interface and analysed using pClamp 9.0 software (all Axon Instruments/Molecular Devices) or WinWCP (Strathclyde Electrophysiological Software) running on a standard PC. For comparisons between muscles and fibres, EPP and MEPP amplitudes were corrected to a standard resting potential of −80 mV. For quantal analysis, EPP amplitudes were corrected for nonlinear summation using the formula [41], *V*′ = *v*/(1 − *fv*/*E*) applying an ‘*f*’ factor of 0.8 and assuming a reversal potential for AChRs of 0 mV. Quantal content was then calculated using the direct method, dividing the corrected EPP amplitude by the mean MEPP amplitude corrected to the EPP resting potential.

### 2.6. Data collection and statistical analysis

All data were exported from ImageJ and collated on a single spreadsheet designed in Microsoft Excel (NMJ-morph template spreadsheets are freely available for download from http://dx.doi.org/10.7488/ds/1490). Spreadsheets were manually checked for numerical errors following data import from ImageJ. Statistical analyses were performed using GraphPad Prism and SPSS software. Individual statistical methods are detailed in the Results section and figure legends.

## 3. Results

To generate experimental preparations of NMJs suitable for performing comparative morphometric analysis, we used immunohistochemically labelled muscle preparations from young adult (approx. two months old) wild-type mice. NMJs were labelled using antibodies against neurofilament (2H3) and synaptic vesicle (SV2) proteins to reveal pre-synaptic neuronal architecture, and α-bungarotoxin to reveal post-synaptic motor endplates [26,42–45] (figure 1). Initial qualitative comparisons of NMJs from three different muscles from the same mouse—interscutularis (IS); triceps brachii (TRI); 4th deep lumbrical from the hindlimb (HL4)—revealed strikingly different synaptic morphologies between the three pools of synapses, with marked differences in the size, appearance and complexity of both pre- and post-synaptic structures (figure 1).

### 3.1. Developing NMJ-morph as a standardized platform for obtaining morphological data

To further explore these variations in NMJ morphology, and facilitate quantitative comparisons and subsequent statistical analyses, we developed NMJ-morph as a standardized workflow through which we could acquire morphological reference data for 21 separate pre- and post-synaptic variables (see Material and methods).

**Figure 3.**
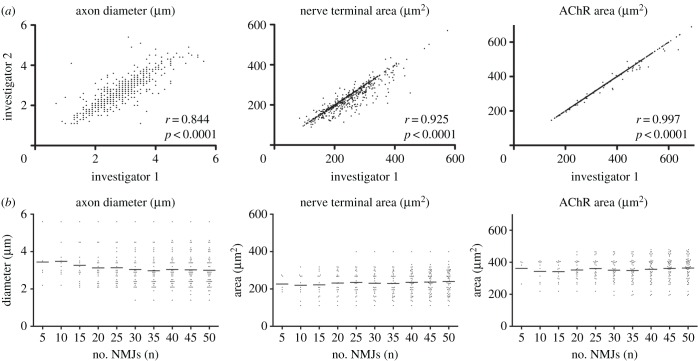
Reliability of NMJ-morph. Two independent investigators used NMJ-morph to analyse a series of 600 NMJs from the 1st deep lumbrical of the hindlimb (6 mice: 3 male, 3 female; 50 NMJs from the lumbrical of each side). (*a*) Strong concordance was demonstrated between the two investigators for all variables. The scatterplots demonstrate the range in correlation, from weakest (axon diameter) to strongest (AChR area). For all variables, the inter-observer correlation was both high (*r* ≥ 0.844) and statistically significant (*p* < 0.0001). (*b*) The importance of adequate sampling when reporting mean values for NMJ variables. The plots correspond to the variables illustrated in (*a*), and show data obtained from a single muscle (50 NMJs). Increasing the sample size results in a gradual plateauing of the mean; on this basis, we recommend a sample of 40 NMJs per muscle to ensure accuracy of reported mean values.

Initially, to determine the reliability of NMJ-morph and identify the degree of any inter-user variability, a series of 600 NMJs were analysed by two independent investigators undertaking measurements on two different workstations (figure 3). In this exercise, investigator 1 selected the *Huang* threshold for 83% of the images (14% manual, 3% *Yen*), while investigator 2 selected *Huang* for 74% (22% manual, 4% *Yen*); overall, the two investigators selected the same threshold (either *Huang, Yen* or manual) for 77% of the images. Despite these variations in the choice of threshold, along with the manual nature of some of the measurements within NMJ-morph, concordance between the two investigators was strong across all NMJ variables: correlation coefficients (*r*) ranged from 0.844 (axon diameter) to 0.997 (AChR area), with *p* < 0.0001 for all variables. As the effect of inter-observer variability attributable to this combination of thresholding and manual measurement is therefore clearly small, we concluded that the outlined workflow was sufficiently robust to be used for the remainder of the study (and recommended for use in general). This initial analysis was also used to identify adequate sample sizes required for accurate assessment of synaptic morphology in NMJ-morph, with a sample size of more than 30 NMJs per muscle being necessary to obtain robust data (figure 3). Based on this observation, all subsequent analyses were undertaken on a sample size of 40 NMJs per muscle.

During the initial development and testing of NMJ-morph, which used both male and female mice from the litter, no overt differences attributable to sexual dimorphism were noted (data not shown). All of the subsequent analysis was therefore performed on a single sex (an arbitrary choice of females), both for consistency of approach and to simplify the (nevertheless significant) problems posed by large-volume data processing.

### 3.2. Baseline morphological data for the mouse neuromuscular junction

**Figure 4.**
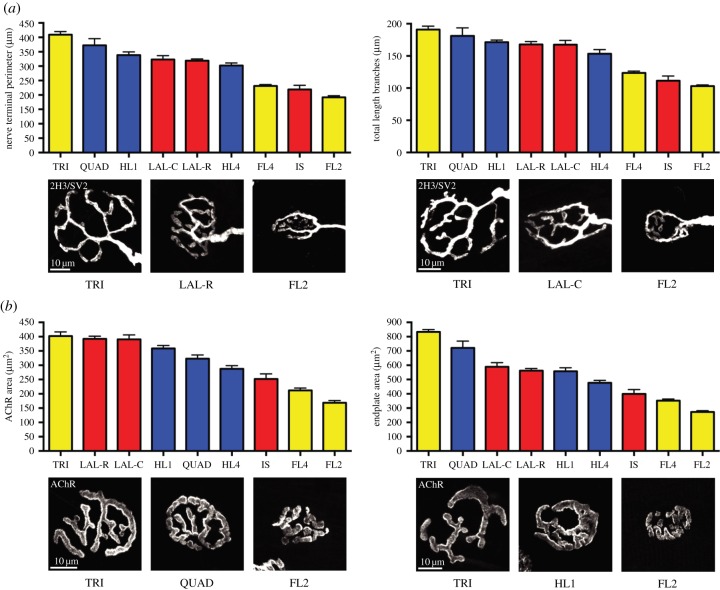
Baseline morphological data for the mouse NMJ. Bar charts demonstrating the variation in NMJ morphology between different muscles, with no clear relationship to body region. (*a*) Pre- and (*b*) post-synaptic examples are shown. Columns depict the mean and standard error of the mean (s.e.m.), calculated from the pooled data of three left/right muscle pairs (240 NMJs). Muscles are ranked according to size and colour-coded to body region (head and neck in red, forelimb in yellow, hindlimb in blue). The example NMJs provide an accurate visual representation of the mean values depicted on the bar chart (to within ±5% of the mean), and correspond to the maximum, median and minimum values. Scale bar = 10 µm. Abbreviations: IS, interscutularis; LAL-R, levator auris longus (rostral band); LAL-C, levator auris longus (caudal band); TRI, triceps brachii; FL2, 2nd deep lumbrical (forelimb); FL4, 4th deep lumbrical (forelimb); QUAD, quadriceps femoris; HL1, 1st deep lumbrical (hindlimb); HL4, 4th deep lumbrical (hindlimb).

Having established that NMJ-morph could be used to generate robust morphological data, we next used this approach to obtain baseline reference data for NMJs from a range of muscles obtained from multiple mice derived from a single litter. Muscles were selected to encompass a broad range of criteria, anticipating that any differences in NMJ morphology might relate directly to these factors. These criteria included coverage of all body regions, proximal/distal position and functional homology within the limbs, and differing nerve supplies, gross architecture and function. The muscles also needed to be in routine biological use. For the purposes of this study, the whole bulk of the quadriceps/triceps (each with a single nerve supply) was treated as a single discrete ‘muscle’, while the individual fore- and hindlimb lumbricals that were selected (each with a different nerve supply) were treated as separate ‘muscles’. Thus, the selections from limbs constituted six discrete muscles (with their six different nerve supplies), rather than two functionally similar groups (quadriceps/triceps and lumbricals). In total, nine anatomically distinct muscles were selected from three different body regions (cranial, forelimbs and hindlimbs) (figure 4).

In keeping with our initial qualitative observations (figure 1), quantitative analyses confirmed marked heterogeneity in NMJ morphology between different muscles (table 1). Significant differences were noted in the mean values across a range of pre- and post-synaptic variables for different muscles (figure 4 and table 1; electronic supplementary material, figure S4). Maximum and minimum values, as well as fold-change differences between the top-ranked and bottom-ranked muscle for each variable, are shown in table 1. The morphological variable with the greatest difference between muscles was average area of AChR clusters, with a fold-change of 3.17 between the top-ranked and bottom-ranked muscle (table 1). By contrast, the variables showing the smallest fold differences were ‘number of axonal inputs’ (not surprisingly, as all NMJs examined were from healthy adult mice, wherein mono-innervation of endplates is to be expected) and ‘overlap’ (table 1; electronic supplementary material, figure S4B). This small difference in ‘overlap’ across the NMJs of different muscles implies that physical congruence between the pre- and post-synaptic components of the NMJ is highly conserved across all synapses, regardless of their individual position or anatomical ‘identity’. Given this high degree of physical congruence between pre- and post-synaptic structures, and the implication that *all* other NMJ variables should be ‘size matched’ across the synapse, there was a striking *lack* of absolute matching nor any clear grouping of NMJ variables by body region or muscle fibre type (note the different orders of muscles in figure 4, both in general and with respect to the pre- and post-synaptic variables). For example, NMJs innervating predominantly fast-twitch muscles could be found with morphological variables at either extreme of the spectrum (e.g. compare FL2 and LAL-R in figure 4). Likewise, NMJs innervating muscles in the forelimb (yellow bars in figure 4) could be found with morphological variables at either end of the range.

In addition to differences in NMJ morphological variables between muscles, the current dataset also reveals heterogeneity within the sets of NMJs from a single muscle sample (i.e. from one side of a single animal). For each such set of 40 NMJs, we calculated the coefficient of variation (CV) for each of the 11 core morphological variables: the median CV was 0.3, with the central 90% of CVs having values between 0.16 and 0.67.

### 3.3. Identifying morphological principles governing synaptic heterogeneity at the neuromuscular junction

Given the apparent lack of obvious predictors of NMJ morphology based on body position or muscle fibre type, we applied more advanced statistical analyses in an attempt to reveal morphological principles that govern synaptic heterogeneity, which were not readily apparent by simple observation. To this end, we performed a principal components analysis (PCA) on the complete multivariate dataset, comprising the values of the 11 ‘core’ morphological variables assessed at each of the 2160 individual NMJs. By systematically assessing correlations between variables, PCA identifies the smallest number of factors (‘principal components’) that account for the greatest degree of variability within the dataset; in what is an ostensibly high-dimensional dataset, this approach enables us to visualize ‘order’ in just two or three dimensions.

**Figure 5.**
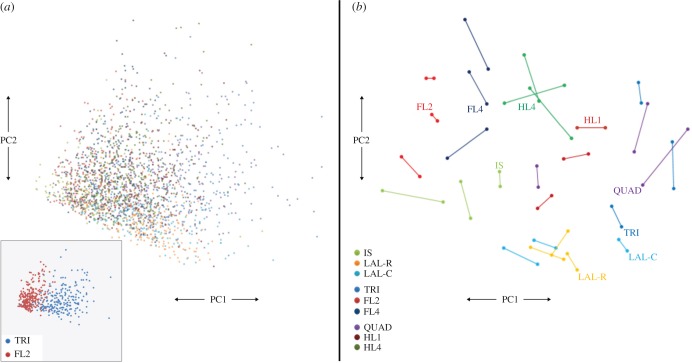
Principal components analysis of NMJs. Two-dimensional PCA representations of the NMJs as defined by the 11 ‘core’ morphological variables. (*a*) PCA map of the complete set of 2160 NMJs, colour-coded by muscle type. The *x*-axis represents the first principal component (−3 to +5), the *y*-axis the second principal component (−5 to +5). The inset shows the same map, but restricted to just NMJs from TRI and FL2 muscles, illustrating the ability of PCA to separate morphologically distinct NMJs. (*b*) PCA map derived from that in panel (*a*), aggregating over the NMJs from each animal. Each data-point represents the centroid of the map positions of the 40 NMJs of a particular muscle from one side of an individual animal; the left and right data-points for each animal are connected by lines. Muscles coloured as in panel (*a*). One line for each muscle type is labelled to aid visual interpretation. PC1 and PC2 coordinates run from −1.5 to +1.5. PCA performed using SPSS.

PCA demonstrated that the majority of the variance (81%) within the dataset could be captured in a two-dimensional plot (figure 5), with the first principal component (PC1) accounting for approximately 72% of the variance, and the second principal component (PC2) accounting for a further approximately 9% of the variance. In physical terms, PC1 is best considered as a reflection of the ‘overall size’ of the NMJ, on the basis of the markedly positive correlations between the size-related morphological variables (electronic supplementary material, figure S5) and their clustering with high *x* coordinate values on the PCA loading plot (electronic supplementary material, figure S6). In a similar manner, PC2 can be interpreted as reflecting the degree of ‘fragmentation’ of the endplate, based on the much weaker association observed between the size-related variables and the number of AChR clusters (electronic supplementary material, figure S5) and the distinctively high *y* coordinate of the number of AChR clusters datapoint on the PCA loading plot (electronic supplementary material, figure S6). A simple way of illustrating how these principal components relate to the measured variables is to regard the components as a ‘summary’ of the variables. A simple analogy is to consider natural variation in animal skeletons—any number of individual variables can be measured (e.g. skull dimensions: height, length, breadth, circumference, weight, etc.), but many of these will be correlated and a large proportion of the overall variability may be found to rest on a much smaller number of principal components (‘size of skull’, ‘shape of skull’, etc.) [46].

The conclusion to be drawn from the PCA is that morphological heterogeneity is determined principally by ‘overall size’ of the NMJ (as opposed to any individual variable), with ‘fragmentation’ of the endplate also making a small but significant contribution to this variation. Equally, no single morphological variable can be used to predict overall NMJ morphology in a given muscle. These two principal components (‘overall size’ and ‘fragmentation’) can be clearly appreciated in the different NMJs depicted in figure 1.

### 3.4. Influence of pre- and post-synaptic cells on neuromuscular junction morphology

**Figure 6.**
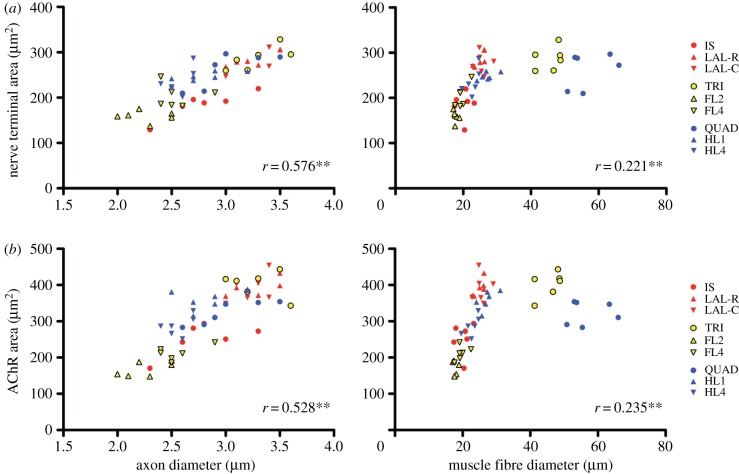
Influence of pre- and post-synaptic cells on NMJ morphology. Scatter plots demonstrating the correlation between axon diameter (or muscle fibre diameter) and individual NMJ variables. (*a*) Pre- and (*b*) post-synaptic examples are shown. To aid visual interpretation, each data-point depicts the mean value over the set of 40 NMJs analysed for a given muscle from one side of a particular animal, but the correlation coefficients have been obtained using the complete set of individual NMJs. Axon diameter demonstrated stronger correlation with the majority of NMJ variables. Muscle fibre diameter showed a nonlinear relationship to the NMJ variables, with muscles grouped in two subsets (see Results). See figure 4 for key to abbreviations and colour-coding.

We next wanted to assess the relative contribution of pre- and post-synaptic cells (represented by axon diameter and muscle fibre diameter, respectively) to the regulation of NMJ morphology. In particular, we wanted to address the tacit assumption that overall NMJ size (identified as a key determinant of synaptic morphology; see above) was simply a consequence of the size of the skeletal muscle fibre on which the NMJ is formed (based on previous links between endplate size and muscle fibre diameter [23,40,47]). We plotted each of the NMJ variables against both axon and muscle fibre diameter individually, and obtained the correlation coefficient and associated *p-*value for each pairing (figure 6 and table 2). Correlation coefficients were of greater magnitude for axon diameter than muscle fibre diameter for the majority of the variables (13 out of 18), with correlations stronger for the ‘core variables’ (similar results were found using Spearman correlation; data not shown). These results suggest that morphological characteristics of the pre-synaptic cell (motor neuron) are a stronger determinant of overall synaptic morphology than those of the post-synaptic cell (muscle fibre).

**Table 2.** Influence of pre- and post-synaptic cells on NMJ morphology. PCA correlation coefficients (*r*) obtained from analysing the complete dataset (2160 NMJs), demonstrating the influence of axon and muscle fibre diameter on each of the NMJ variables. The coefficients in bold type indicate the stronger correlation between axon diameter and the respective variables. Significance levels: **p < 0.01, *p < 0.05.

	correlation with axon diameter	correlation with muscle fibre diameter
core variables		
*pre-synaptic*		
(1) nerve terminal area (µm^2^)	0.576**	0.221**
(2) nerve terminal perimeter (µm)	0.433**	0.387**
(3) number terminal branches	0.198**	0.293**
(4) number branch points	0.303**	0.066**
(5) total length branches (µm)	0.471**	0.311**
*post-synaptic*		
(6) AChR area (µm^2^)	0.528**	0.235**
(7) AChR perimeter (µm)	0.376**	0.420**
(8) endplate area (µm^2^)	0.467**	0.457**
(9) endplate perimeter (µm)	0.429**	0.474**
(10) endplate diameter (µm)	0.449**	0.429**
(11) number AChR clusters	0.057**	0.017
derived variables		
*pre-synaptic*		
(12) average length branches (µm)	0.110**	−0.051*
(13) complexity	0.349**	0.205**
*post-synaptic*		
(14) average area AChR clusters (µm^2^)	0.185**	0.029
(15) fragmentation	−0.070**	−0.046*
(16) compactness (%)	−0.010**	−0.460
(17) overlap (%)	0.065**	−0.220**
(18) area of synaptic contact (µm^2^)	0.585**	0.140**

Interestingly, whereas the relationship between axon diameter and NMJ morphology was linear across the entire range of axon diameters observed, the relationship between muscle fibre diameter and NMJ morphology was quite different: the initially strong linear relationship observed for small diameter muscle fibres did not hold for the larger diameter muscle fibres of triceps and quadriceps (figure 6). Thus, NMJs from triceps and quadriceps were smaller (based on both nerve terminal area and AChR area measurements) relative to their muscle fibre diameters than would be predicted by extrapolating the relationship observed in the cranial and lumbrical muscles. These observations further challenge the notion that NMJ size is a simple function of muscle fibre diameter. Whether or not the closer correlation with axon diameter implies a greater influence of the pre-synaptic cell on NMJ morphology remains to be determined. For example, retrograde signalling from the muscle fibre might still influence NMJ morphology, despite the weaker size correlation. The discrepancy between the predicted and observed sizes of NMJs on the larger muscle fibres (in triceps and quadriceps) might be explained by a volume limitation of the pre-synaptic neuronal cytoplasm.

It is also of note that the observed relationship between muscle fibre diameter and NMJ ‘size’ (as described above) was not influenced by the size of the individual animals; no correlation was found either between animal ‘size’ (weight) and NMJ ‘size’, nor between animal ‘size’ (weight) and muscle fibre diameter.

### 3.5. Within-individual regulation of synaptic morphology

The marked heterogeneity in NMJ morphology that we observed between different muscles led us to question the extent to which synaptic morphology was controlled within a single population of synapses (e.g. all NMJs within an anatomically defined muscle), between the two sides of the same individual (e.g. comparing NMJs in the same muscle on the left and right side of the body), and between different individuals (e.g. comparing NMJs in the same muscle between different mice from a single litter).

**Figure 7.**
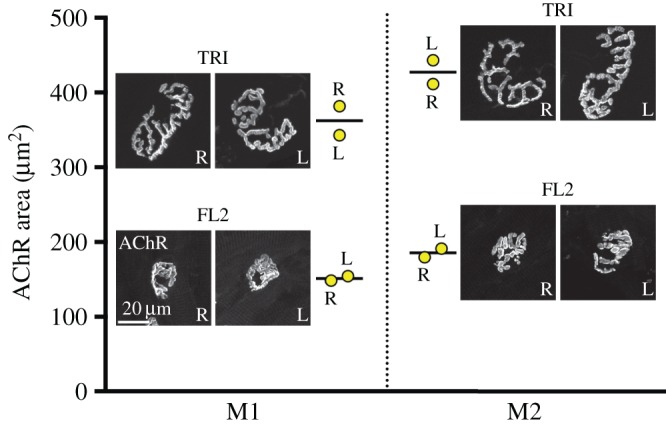
Influence of side, muscle and individual on NMJ morphology. Comparison of left/right pairs of two muscles (TRI and FL2) from two littermate mice (M1 and M2), with respect to mean AChR area of the NMJ. Each data-point in yellow (an individual muscle, left or right) represents the mean of 40 NMJs; the mean of the left/right pair is indicated by the line. Representative pairs of NMJs are shown alongside. Scale bar = 20 µm.

Figure 7 demonstrates a systematic approach to pairwise comparison of these factors (muscle, side and mouse) by displaying the measurements for one ‘core’ NMJ variable (AChR area) for left/right pairs of two different forelimb muscles (TRI and FL2) in two littermate mice. In this example, it is immediately apparent that the greatest differences in AChR area are observed in pairwise comparison of TRI versus FL2 in each mouse, followed next by between-mouse comparisons (i.e. M1 v. M2 for each muscle, either TRI or FL2), with the comparison between equivalent muscles on the left/right side of the body showing the smallest differences.

To quantify the influence of each of these factors for all muscles across the whole litter, we performed a three-way analysis of variance (ANOVA) on the complete dataset of 2160 individual NMJs (table 3). This confirmed that the largest influence on NMJ morphology came from the ‘identity’ of the muscle (i.e. the largest differences were observed when comparing the morphology of NMJs between different individual muscles, regardless of side or the individual ‘identity’ of the animal).

**Table 3.** Influence of side, muscle and individual on NMJ morphology. Significance levels obtained from three-way analysis of variance (ANOVA), demonstrating the influence of each factor on the natural variation of NMJ morphology. Muscle identity has the greatest influence; side has no effect. Significance levels: ***p < 0.001, **p < 0.01, *p < 0.05. ANOVA performed using SPSS.

	muscle	mouse	side
core variables			
*pre-synaptic*			
(1) nerve terminal area (µm^2^)	***		
(2) nerve terminal perimeter (µm)	***		
(3) number terminal branches	***	**	
(4) number branch points	**		
(5) total length branches (µm)	***		
*post-synaptic*			
(6) AChR area (µm^2^)	***		
(7) AChR perimeter (µm)	***	*	
(8) endplate area (µm^2^)	***		
(9) endplate perimeter (µm)	***	*	
(10) endplate diameter (µm)	***	*	
(11) number AChR clusters	***		*
derived variables			
*pre-synaptic*			
(12) average length branches (µm)	*	**	
(13) complexity	***		
*post-synaptic*			
(14) average area AChR clusters (µm^2^)	***		
(15) fragmentation	**		
(16) compactness (%)	***	*	
(17) overlap (%)	**	*	
(18) area of synaptic contact (µm^2^)	***		
associated nerve & muscle variables			
(19) axon diameter (µm)	***		
(20) muscle fibre diameter (µm)	***		

It is conceivable that the absence of left/right differences in our analyses reported in table 3 was the result of pooling of data from individual muscles and animals, with the possibility that this pooling masked genuine left/right differences within individual animals. We tested for this eventuality by performing a series of *t*-tests comparing the mean values of a particular variable (AChR area) for the NMJs of each left/right muscle pair in each individual mouse. In this set of 27 (=3 × 9) left/right comparisons, only one showed a statistically significant (*p* < 0.05) difference (after Bonferroni correction). An equivalent series of left/right comparisons for the derived variable ‘fragmentation’ also failed to demonstrate any statistically significant differences (after Bonferroni correction; electronic supplementary material, figure S7). These analyses confirmed a consistent replication of average NMJ morphology across left/right pairs of each muscle in the animal, which held true for each individual in the litter studied; this conservation of morphology between sides (table 3) accounts for the small error bars noted for each of the individual muscles shown in figure 4 (electronic supplementary material, figure S4).

### 3.6. Influence of morphological variables on synaptic function at the neuromuscular junction

**Figure 8.**
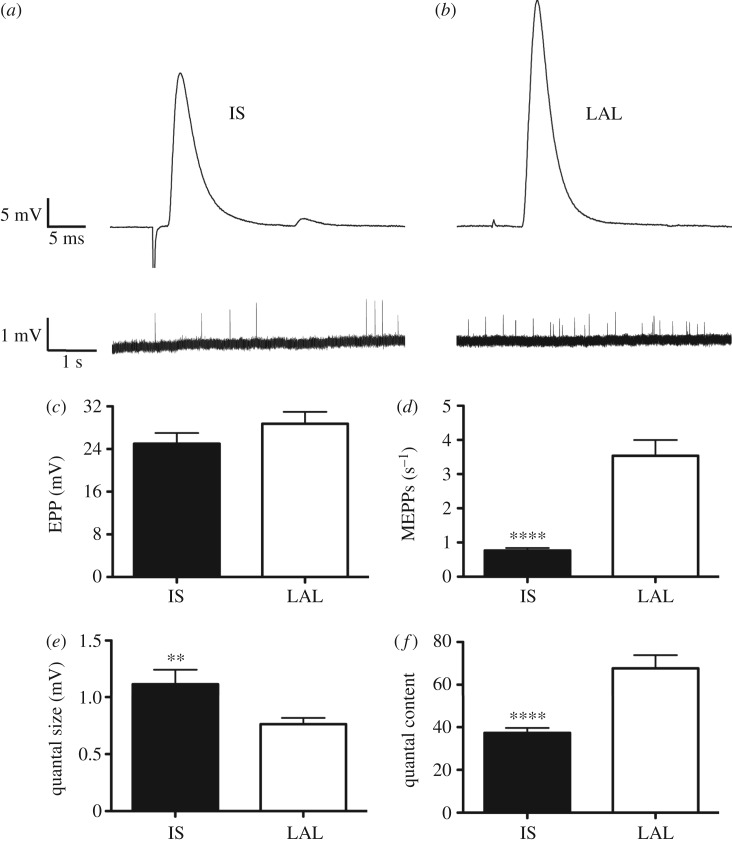
Electrophysiological analysis of NMJs in IS and LAL. These findings supported the observed morphological differences (figure 4) and were consistent with previous findings regarding the relationship between nerve terminal size and synaptic strength. (*a*,*b*) EPPs (upper traces) and spontaneous MEPPs (lower traces; higher gain, slower time base) recorded from representative (*a*) IS and (*b*) LAL muscle fibres. (*c*) There was no significant difference in EPP amplitude comparing the two muscles. (*d*–*f*) Spontaneous MEPP frequency was about four times lower in IS muscle fibres compared with LAL (*d*). Conversely, MEPP amplitude (quantal size) was about 45% greater in IS than LAL fibres (*e*). Quantal content was about 87% higher in LAL compared with IS (*f*), offsetting the difference in quantal size and thus accommodating nonlinear summation and conferring EPP amplitude and safety factor for transmission at equivalent levels. Data are mean ± SEM; *n* = 33–58 muscle fibres recorded in *N* = 2 IS and *N* = 3 LAL muscles; **p < 0.01; ****p < 0.0001; unpaired *t*-tests).

Finally, we asked to what extent the range of pre- and post-synaptic morphologies observed in our experiments had an impact on the functional capacity of NMJs. The relationship between structure and function is critical for preserving the high ‘safety factor’ at the NMJ [17]—the ability to release sufficient neurotransmitter to elicit an action potential in the muscle fibre [48,49]. For these experiments, we performed electrophysiological analyses of NMJs in the IS and LAL muscles, selected on the basis of their neighbouring positions in the body (receiving a nerve supply from the same major peripheral nerve) but with systematic and significantly different NMJ morphologies (figures 4 and 8, and tables 1 and 4; electronic supplementary material, figure S4).

**Table 4.** Electrophysiological properties of IS and LAL. Summary of electrophysiological properties of IS and LAL muscles. Values are the mean and standard error of the mean (s.e.m.) for each variable, and the fold difference between the means for each muscle. Data recorded from 33–58 muscle fibres in two IS muscles and three LAL muscles. Significance levels: ****p < 0.0001, **p < 0.01, *p < 0.05.

	IS	LAL	fold difference
resting membrane potential (mV)	−64.0 ± 1.00	−60.0 ± 1.00	1.07*
EPP amplitude (mV)	25.0 ± 2.00	28.7 ± 2.30	1.15
EPP rise time (ms)	1.12 ± 0.06	0.82 ± 0.05	1.37****
EPP decay time (ms)	2.05 ± 0.08	1.36 ± 0.05	1.51****
MEPP frequency (s^−1^)	0.77 ± 0.07	3.54 ± 0.46	4.60****
MEPP amplitude (mV)	1.12 ± 0.13	0.76 ± 0.06	1.47**
quantal content	37.3 ± 2.40	67.7 ± 6.20	1.82****

Resting membrane potentials in IS (−64 ± 1 mV, mean ± s.e.m., *n* = 40 fibres, *N* = 2 muscles) were slightly (by about 2–6 mV) more negative than in LAL (−60 ± 1 mV, *p* < 0.03, *t*-test). EPP amplitudes (corrected to a resting potential of −80 mV) in IS (25.0 ± 2.0 mV, *n* = 40 fibres, *N* = 2 muscles) and LAL (28.7 ± 2.3 mV, *n* = 56 fibres, *N* = 3 muscles) were not significantly different (*p* = 0.24). EPP rise and decay times were significantly longer in IS (1.12 ± 0.06 ms, 2.05 ± 0.08 ms, respectively, *n* = 50 fibres, *N* = 2 muscles) than in LAL (0.82 ± 0.05 ms, 1.36 ± 0.05 ms, *n* = 58 fibres, *N* = 3 muscles, *p* < 0.0001). These data predict a lower membrane resistance and shorter membrane time constant in LAL than IS muscle fibres.

MEPP frequency at the NMJ was more than four times lower in IS muscles (0.77 ± 0.07 s^−1^, *n* = 34 fibres, *N* = 2 muscles) than in LAL (3.54 ± 0.46 s^−1^, *n* = 46 fibres, *N* = 3 muscles, *p* < 0.0001). These data are consistent with observations of higher MEPP frequencies at larger endplates made previously [23]. Likewise, MEPP amplitude (quantal size), corrected to a standard resting membrane potential of −80 mV, was about 45% greater in IS (1.12 ± 0.13 mV, *n* = 33 fibres, *N* = 2 muscles) than in LAL (0.76 mV ± 0.06 mV, *n* = 47 fibres, *N* = 3 muscles, *p* < 0.01), consistent with previous reports [23]. The mean quantal content of the initial EPPs, calculated after correction of EPP amplitudes for nonlinear summation and resting potential, was about 85% larger in LAL (67.7 ± 6.2 quanta, *n* = 45 fibres, *N* = 3 muscles) than in IS (37.3 ± 2.4 quanta, *n* = 33 fibres, *N* = 2 muscles, *p* < 0.0001), consistent with the well-established correlation between quantal content and nerve terminal area at NMJs [23,38–40,49,50].

Taken together, our physiological measurements are consistent with previous findings [23,38–40,49,50] regarding the relationship between synaptic size and synaptic strength and further validate the morphometric differences between anatomically defined muscles that we have reported here.

## 4. Discussion

Here, we report on the successful development and implementation of an ImageJ-based platform/workflow to facilitate comparative morphometric analyses of the NMJ (NMJ-morph). Using NMJ-morph, we have generated baseline reference data for 21 individual pre- and post-synaptic morphological variables at the NMJ, revealing systematic differences in NMJ morphology between defined synaptic populations. More detailed statistical interrogation of these data using PCA revealed overall size of NMJ and degree of synaptic fragmentation to be the most critical parameters in defining synaptic morphology. We also demonstrate that ‘average’ synaptic morphology is highly conserved between NMJs located in the same anatomically discrete muscles on the left and right sides of the body, and furthermore confirm that systematic differences in synaptic morphology predict corresponding differences in synaptic function.

Demonstrating the utility of NMJ-morph as a robust and repeatable methodology for comparative morphometric analysis of NMJs suggests that it will now be possible to apply this approach to a wide variety of experimental settings (including the study of pathological changes occurring at the NMJ during a range of neurodegenerative conditions). The benefits of adopting such a standardized approach include the ability to quickly and easily compare and combine datasets from multiple studies generated by different laboratories (which remains extremely difficult at present, when the current practice of individual laboratories is to use distinct and often subtly different approaches to NMJ morphometrics). By providing free access to a comprehensive NMJ-morph User Guide (describing the step-by-step method of performing each measurement), alongside spreadsheet templates and a sample image bank for use in training and standardization, we anticipate that this approach will be attractive to other laboratories. Moreover, the provision of baseline morphological reference data from numerous different muscle groups in this study should aid in the design and interpretation of future studies of the NMJ in health and disease.

In this study, we have focused on a comprehensive analysis of NMJ morphology in a single litter of CD1 mice. Given the perhaps unexpected degree of NMJ heterogeneity noted between muscles and individual littermate animals, it remains to be seen whether such variation also extends to ‘between-litter’ and ‘between-strain’ comparisons; these analyses warrant further exploration in future research, and we would caution against regarding the values reported here as being ‘normal’ or ‘typical’ for all mice. If high levels of NMJ heterogeneity are indeed present across different strains of mice, this could have significant implications for translational research into synaptic pathology in neurodegenerative disease.

Furthermore, our observation of highly conserved/regulated ‘average’ synaptic morphology at mouse NMJs located in the same muscle but on different sides of the body contrasts with the marked differences in synaptic morphology when comparing NMJs in the same muscle again, but between two individual animals; these observations are in contrast with previous findings from invertebrate model systems. In *Drosophila* for example, quantitative analysis of NMJ morphology on muscle 4 across 20 different sub-species revealed little morphological variation when comparing individual animals within each species, with extensive variation in NMJ size and complexity only occurring between species [10]. These findings suggest that the control of synaptic morphology, which appears to be tightly regulated in *Drosophila* (presumably genetically), is not as highly conserved in the more complex mammalian nervous system. Thus, there is a level of heterogeneity in synaptic morphology to be found in mice that may not be apparent in well-studied invertebrate systems. It may not be possible therefore to simply translate findings concerning the genetic or environmental control of synaptic structure and function obtained from model systems such as *Drosophila* directly across to the mammalian nervous system. However, the availability of NMJ-morph will now facilitate a more direct re-examination of apparent invariability in other species, while in the mouse, the reference data that we have established for healthy NMJs across a range of muscles invites future comparisons with NMJs in the diseased state.

Given the differences observed at the NMJ in mice, it remains to be established whether the morphological principles identified in this study are suitable for extrapolation to other mammalian species, including humans. This may be of considerable importance with respect to studies of neurodegenerative conditions affecting neuromuscular synapses, where the majority of pre-clinical research relies on rodent models of disease. Studies directly addressing the conservation (or otherwise) of regulatory control of synaptic morphology between lower mammals (e.g. mice) and humans are therefore warranted, albeit technically challenging, because previous studies of human NMJs have typically relied on motor point biopsy [51], a once commonly indicated diagnostic procedure which is rarely performed in modern clinical practice.

The electrophysiological data we obtained are consistent with our findings of smaller muscle fibres and smaller NMJs in IS compared with LAL, as quantal content in mammalian muscle fibres is known to be proportional to motor nerve terminal size [23,38–40,49,50]. As a result, relatively small muscle fibres have higher input resistance, longer membrane time constant, and NMJs showing larger MEPPs (quantal size) and lower quantal contents than larger fibres [23,40]. The higher quantal content we observed in LAL (consistent with larger nerve terminals) and lower quantal size (consistent with larger muscle fibres) approximately offset one another, so that EPP amplitude was almost the same as in IS, thereby supporting the notion of a homeostatic mechanism that conserves safety factor for neuromuscular transmission [40,50,52]. It should be noted however that we sampled EPPs blindly, using single microelectrodes; further insights could be obtained by the simultaneous imaging of NMJ morphology alongside electrophysiological recordings from the same muscle fibres (e.g. allowing quantal analyses to be acquired directly from recordings of endplate currents obtained using a two-electrode voltage clamp [41]). Wood & Slater [53,54] measured safety factor in this fashion by comparing the observed endplate current with that required to critically depolarize muscle fibres to the action potential firing threshold. They concluded that, once differences in the contribution of post-synaptic junctional folding were taken into account, the safety factor in rat soleus (a slow-twitch muscle) corresponded to about 3.5 times as much neurotransmitter released at an NMJ over the requirement to trigger muscle action potentials; but this was significantly lower than at NMJs in extensor digitorum longus (a fast-twitch muscle), where the safety factor was about 5. It thus remains unclear exactly which property of the NMJ is homeostatically regulated [52,55], and whether NMJs in IS and LAL muscles normally differ in this regard. Further analysis of endplate currents in these two muscles, taken together with the present observations, may yield novel insights into factors that regulate synaptic depolarization. This method of simultaneous structure–function recording from the same NMJs has been used recently by Willadt *et al.* [56], adding considerably to the scope of questions that can be addressed in future research.

In summary, we have successfully developed a new ImageJ-based workflow (NMJ-morph) to facilitate comparative morphometric analyses of the NMJ. We present baseline reference data for 21 individual pre- and post-synaptic morphological variables at the NMJ that revealed systematic differences between defined synaptic populations. We conclude that the overall size of an NMJ and the degree of synaptic fragmentation are the most critical parameters for defining synaptic morphology *in vivo*.

## Supplementary Material

NMJ-morph reveals principal components of synaptic morphology influencing structure-function relationships at the neuromuscular junction - Supplementary Figures and Tables

## Data accessibility

All experimental data/spreadsheets, research materials (e.g. image banks) and NMJ-morph templates reported in this paper (beyond those incorporated in the figures and electronic supplementary material) will be made freely available upon publication at http://dx.doi.org/10.7488/ds/1490.
