# Correction to ‘NMJ-morph reveals principal components of synaptic morphology influencing structure–function relationships at the neuromuscular junction’

**DOI:** 10.1098/rsob.160335

**Published:** 2017-01-18

**Authors:** Ross A. Jones, Caitlan D. Reich, Kosala N. Dissanayake, Fanney Kristmundsdottir, Gordon S. Findlater, Richard R. Ribchester, Martin W. Simmen, Thomas H. Gillingwater

*Open Biol*. **6**, 160240. (Published online 7 December 2016). (doi:10.1098/rsob.160240)

A correction is required for the manuscript detailed below: in Table 2, the significance levels for variable (16) should read: −0.010 and −0.460**.

