# Supplementary material for: NMJ-morph reveals principal components of synaptic morphology influencing structure–function relationships at the neuromuscular junction
Source: Open Biol. 2016 Dec 7;6(12):160240. doi: 10.1098/rsob.160240 (PMC5204123; doi:10.1098/rsob.160240)
Supplement: NMJ-morph reveals principal components of synaptic morphology influencing structure-function relationships at the neuromuscular junction - Supplementary Figures and Tables [file rsob160240supp1.pdf]

NMJ-morph reveals principal components of synaptic morphology influencing structure-function relationships at the neuromuscular junction

Ross A. Jones, Caitlan D. Reich, Kosala N. Dissanayake,

Fanney Kristmundsdottir, Gordon S. Findlater, Richard R. Ribchester,

Martin W. Simmen & Thomas H. Gillingwater

Open Biology

DOI: 10.1098/rsob.20160240

Supplementary Material

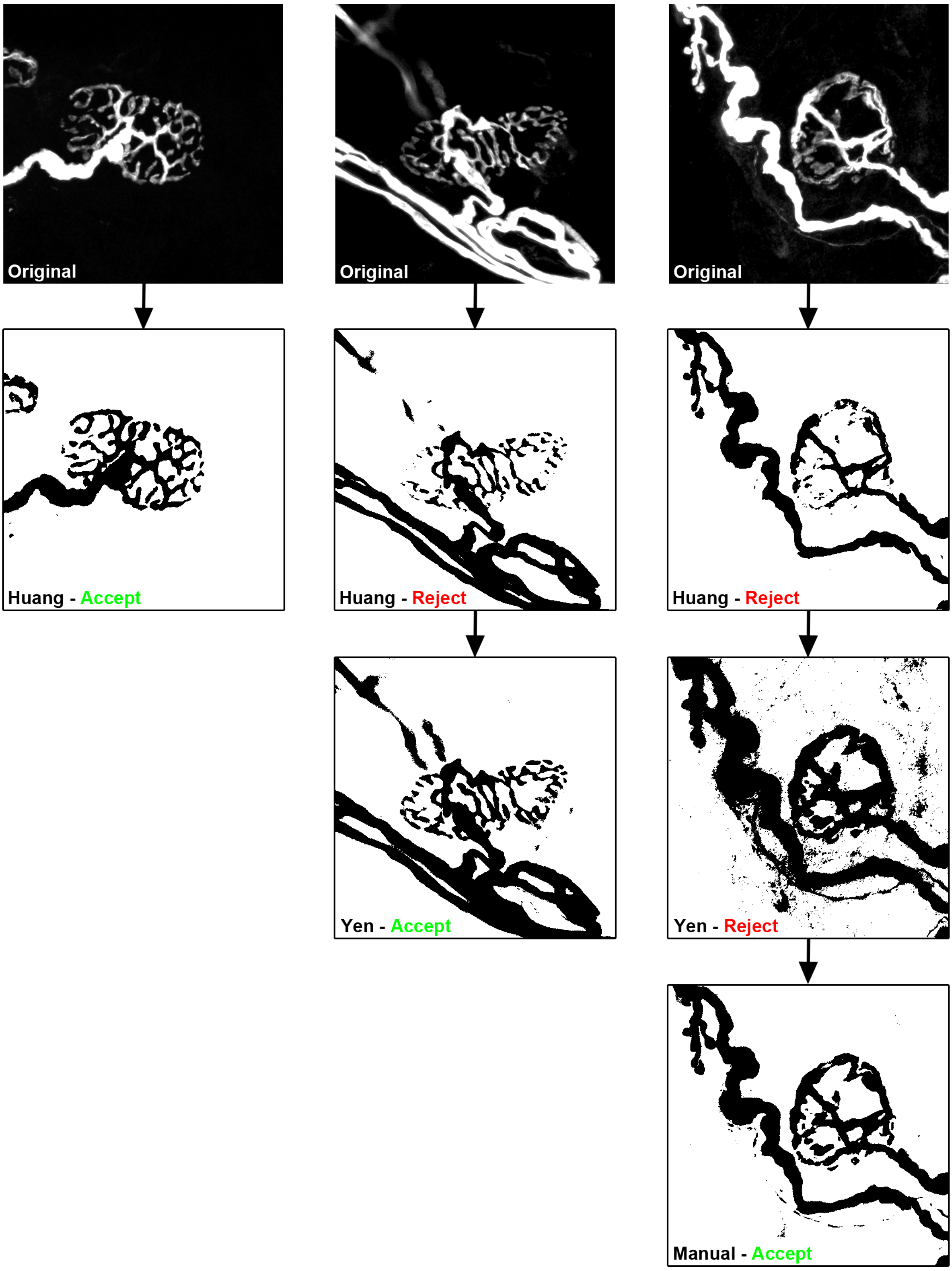

Supplementary Figure 1 | Thresholding of images. Flowchart demonstarting the procedure for accurate thresholding of images. Three examples are shown. Most images (79%) could be accurately thresholded using the *Huang* setting (left panels), a small minority (3%) were better represented with the *Yen* method (middle panels), whilst the remainder of the images (18%) required a manual adjustment of the threshold (right panels).

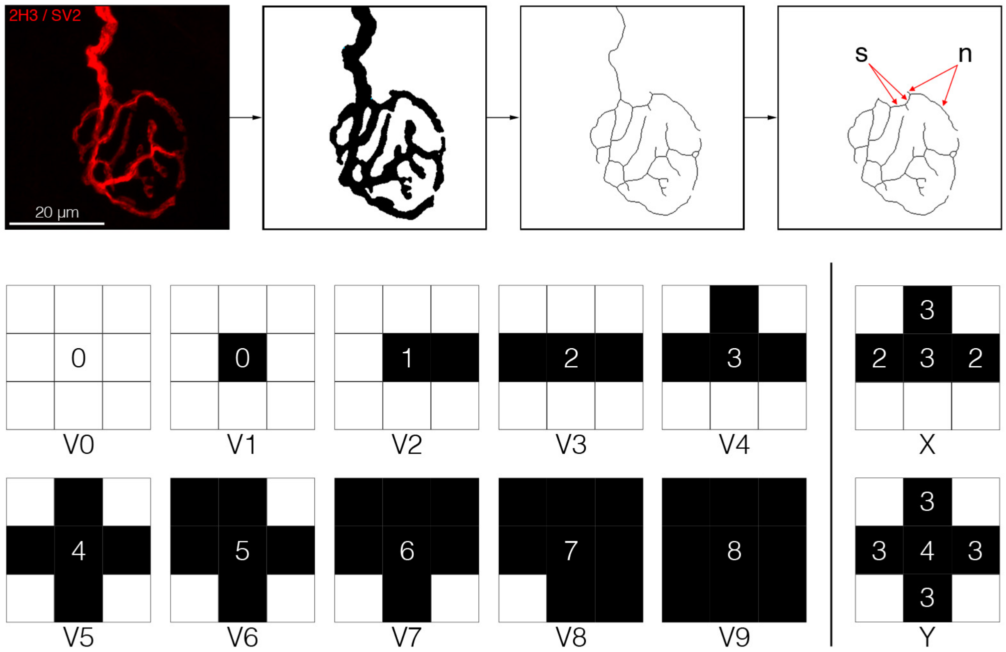

Supplementary Figure 2 | BinaryConnectivity analyses. The ImageJ plugin, BinaryConnectivity, was used to analyze the branching pattern of pre-synaptic 'skeletons'. BinaryConnectivity calculates the total number of each type of pixel in the binary image. The 10 different types of pixel ('values' V0-V9) are shown. The number in the pixel itself refers to the number of neighbouring pixels. Value 0 = a background white pixel. Value 1 = a solitary black pixel. Value 2 = a terminal pixel. Value 3 = a pixel along a branch. Values 4 and 5 = branch pixels (with three and four branches, respectively – the most commonly occurring branch pixels). Values 6-9 = branch pixels (uncommon). The ***number of terminal branches (n)*** in the skeleton is given by the total of the Value 2 pixels. The ***number of branch points*** is given by the sum of the Value 4 and 5 pixels, to which a correction factor is applied in the spreadsheet – this compensates for the 'over-counting' of pixels immediately adjacent to the actual branch points, as shown in X and Y. The ***total length of branches (l)*** is derived from the number of pixels in the skeleton (sum of Values 1-9), and is calculated automatically in the NMJ-morph spreadsheet. Note that the ***average length of branches (l/n)*** is an index rather than a true average, as the length (l) includes both the terminal branches (n) and the intermediate segments (s) (marked on the pre-synaptic 'skeleton'). The derived variable 'complexity' = $\log_{10}$ (no. terminal branches × no. branch points × total length of branches).

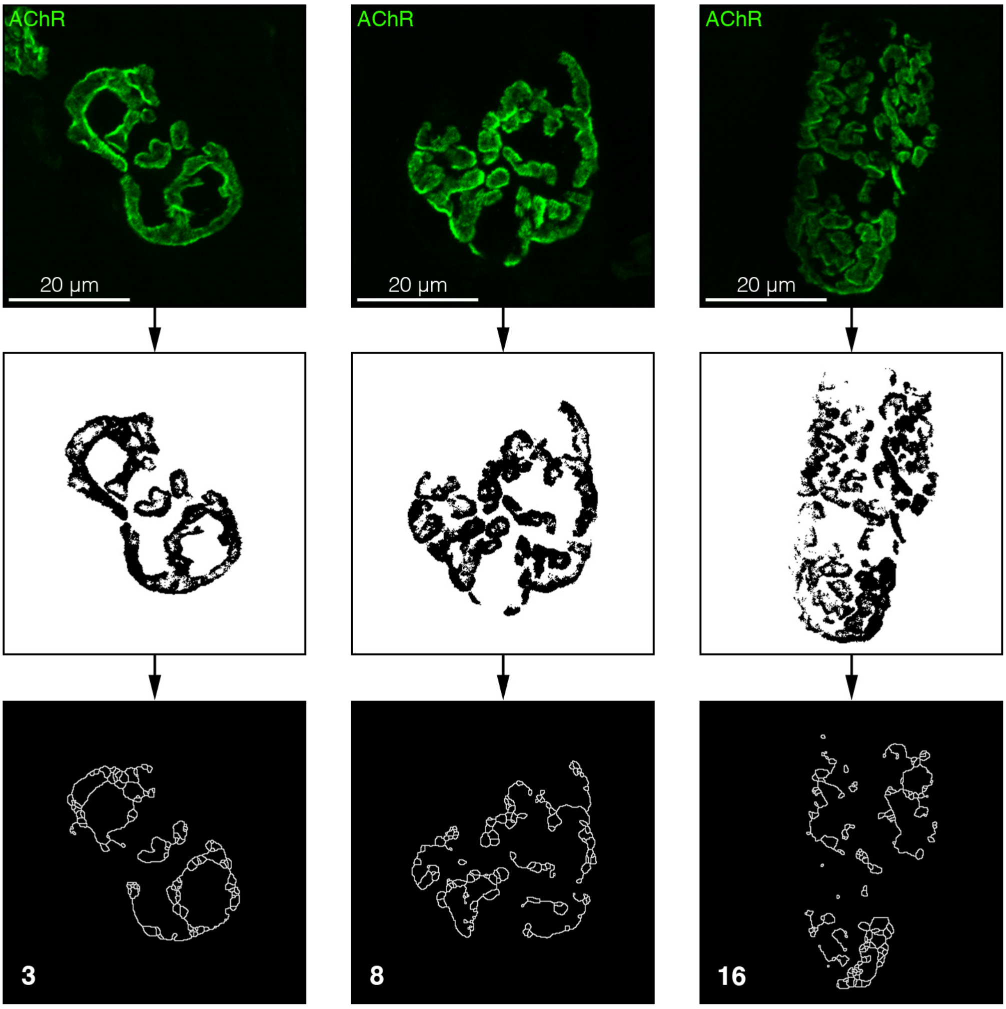

Supplementary Figure 3 | Segmented particles analyses. The segmented particles function in ImageJ is used to count the number of AChR clusters and calculate the endplate 'fragmentation' index. Three endplates have been arranged in order of increasing fragmentation from left to right based on visual assessment (top panels). The original images have been converted directly to binary (middle panels) and the segmented particles function has been applied (bottom panels). The number of discrete 'segmented particle' clusters that are generated (3, 8 and 16 respectively) reflects the increasing degree of fragmentation of the three endplates. The derived variable 'fragmentation' = 1 – (1 / number of AChR clusters).

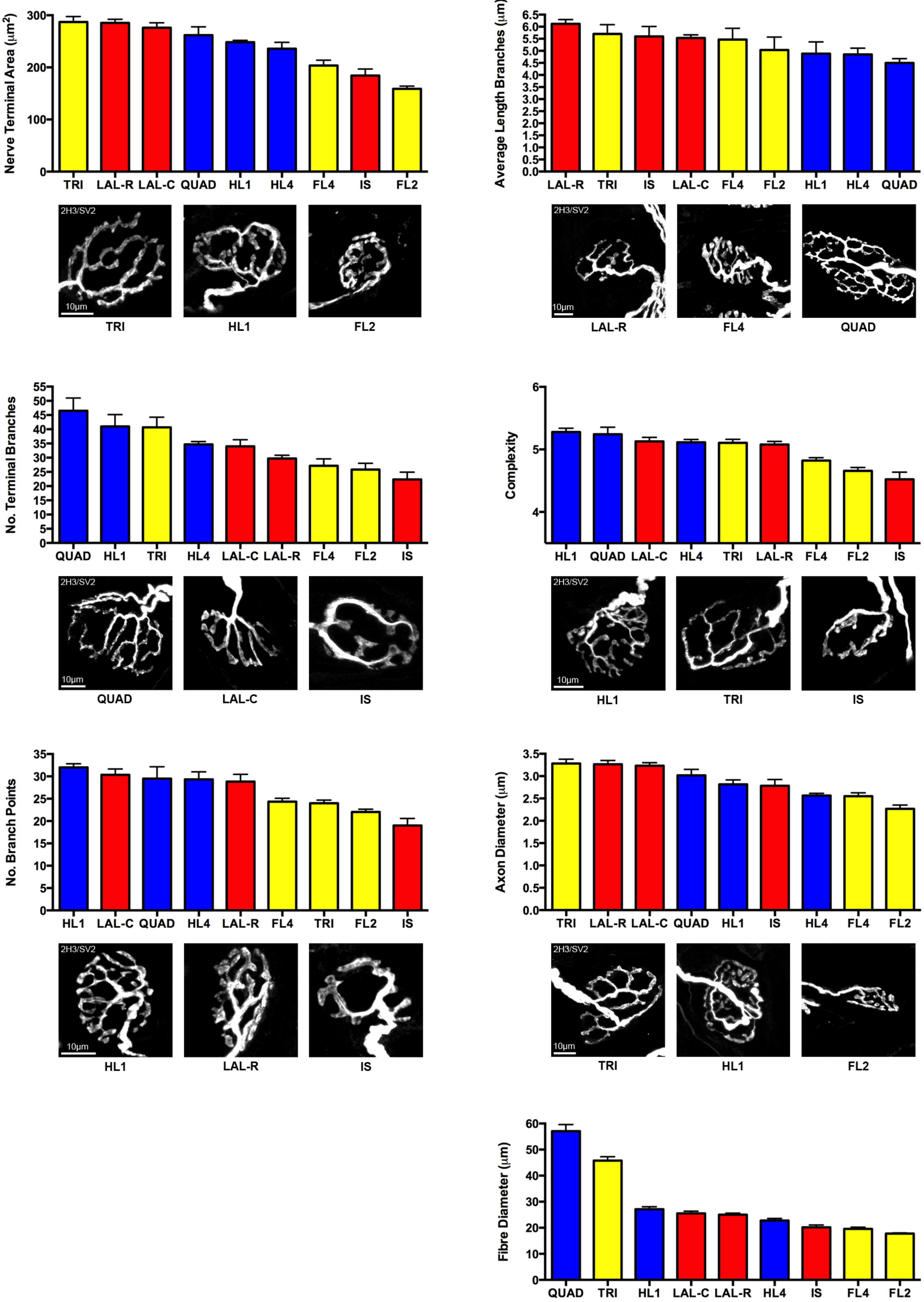

Supplementary Figure 4A | Baseline morphological data for the mouse NMJ. The remaining pre-synaptic variables are shown; the data is represented in the same format as Figure 4. Also included is the ranked bar chart of the muscle fibre diameters.

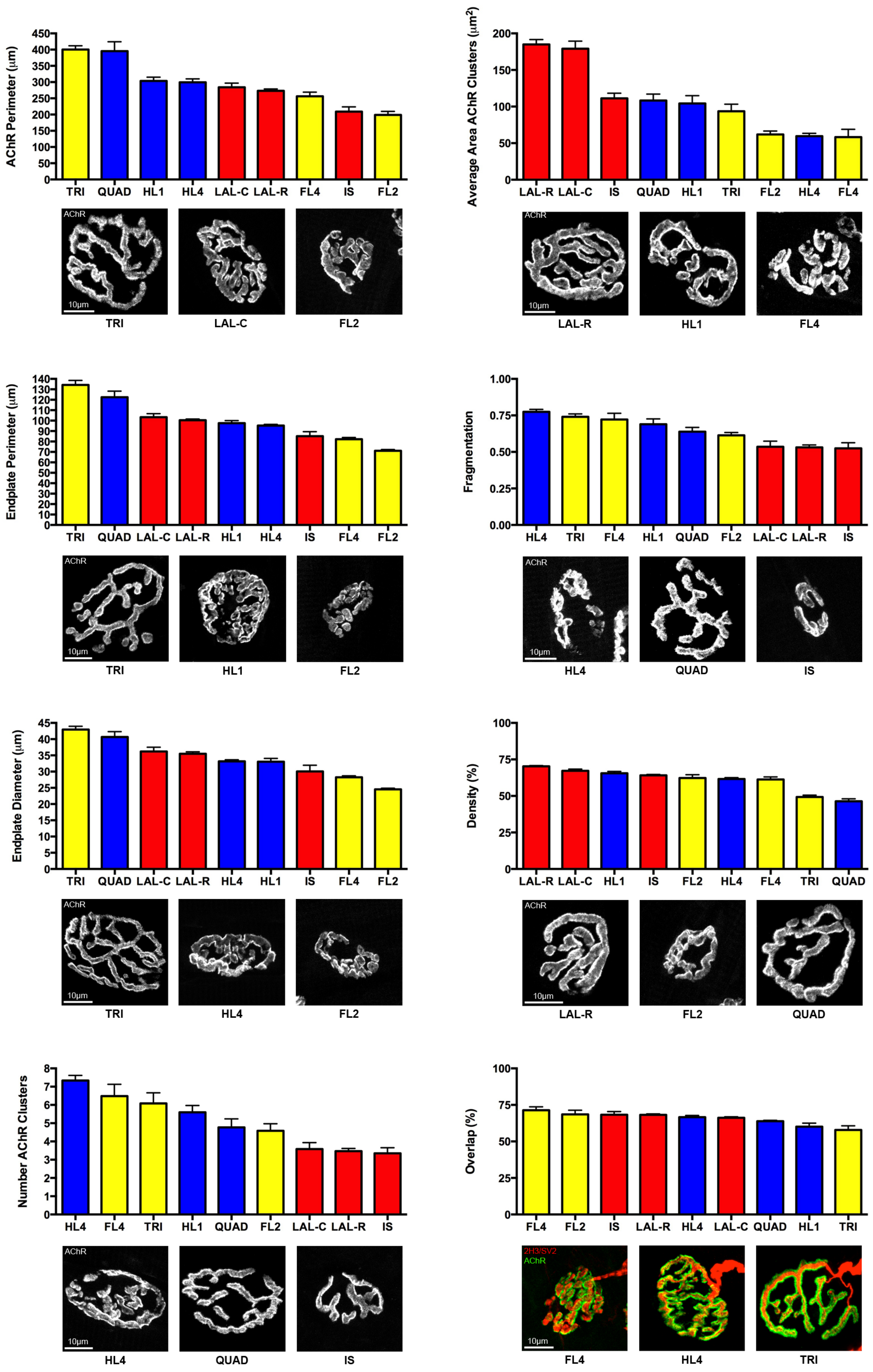

Supplementary Figure 4B | Baseline morphological data for the mouse NMJ. The remaining post-synaptic variables are shown; the data is represented in the same format as Figure 4.

	Nerve terminal area	Nerve terminal perimeter	Number terminal branches	Number branch points	Total length of branches	AChR area	AChR perimeter	Endplate area	Endplate perimeter	Endplate diameter	Number AChR clusters	Average length of branches	Complexity	Average area AChR clusters	Fragmentation	Compactness	Overlap	Area synaptic contact	Axon diameter	Fibre diameter
Nerve terminal area	1	0.836	0.423	0.649	0.897	0.827	0.713	0.794	0.742	0.742	0.25	0.154	0.695	0.175	-0.228	-0.13	0.087	0.905	0.576	0.221
Nerve terminal perimeter	0.836	1	0.739	0.666	0.966	0.848	0.883	0.923	0.872	0.833	0.332	-0.164	0.842	0.107	-0.296	-0.327	-0.345	0.73	0.433	0.387
Number terminal branches	0.423	0.739	1	0.63	0.684	0.536	0.701	0.645	0.617	0.569	0.344	-0.621	0.824	-0.016	-0.282	-0.311	-0.565	0.311	0.198	0.293
Number branch points	0.649	0.666	0.63	1	0.794	0.576	0.526	0.496	0.446	0.461	0.189	-0.162	0.862	0.164	-0.144	0.035	-0.064	0.587	0.303	0.066
Total length of branches	0.897	0.966	0.684	0.794	1	0.847	0.83	0.869	0.812	0.792	0.293	-0.068	0.876	0.155	-0.255	-0.22	-0.2	0.796	0.471	0.311
AChR area	0.827	0.848	0.536	0.576	0.847	1	0.672	0.856	0.747	0.756	0.098	-0.029	0.711	0.342	-0.124	0.039	-0.305	0.905	0.528	0.235
AChR perimeter	0.713	0.883	0.701	0.526	0.83	0.672	1	0.88	0.833	0.784	0.475	-0.192	0.728	-0.081	-0.406	-0.551	-0.292	0.566	0.376	0.42
Endplate area	0.794	0.923	0.645	0.496	0.869	0.856	0.88	1	0.909	0.86	0.292	-0.101	0.699	0.111	-0.272	-0.431	-0.337	0.731	0.467	0.457
Endplate perimeter	0.742	0.872	0.617	0.446	0.812	0.747	0.833	0.909	1	0.91	0.321	-0.103	0.66	0.051	-0.293	-0.457	-0.319	0.632	0.429	0.474
Endplate diameter	0.742	0.833	0.569	0.461	0.792	0.756	0.784	0.86	0.91	1	0.259	-0.073	0.651	0.098	-0.258	-0.376	-0.264	0.671	0.449	0.429
Number AChR clusters	0.25	0.332	0.344	0.189	0.293	0.098	0.475	0.292	0.321	0.259	1	-0.184	0.315	-0.601	-0.715	-0.427	-0.098	0.055	0.057	0.017
Average length of branches	0.154	-0.164	-0.621	-0.162	-0.068	-0.029	-0.192	-0.101	-0.103	-0.073	-0.184	1	-0.398	0.114	0.156	0.174	0.559	0.186	0.11	-0.051
Complexity	0.695	0.842	0.824	0.862	0.876	0.711	0.728	0.699	0.66	0.651	0.315	-0.398	1	0.113	-0.278	-0.153	-0.342	0.611	0.349	0.205
Average area AChR clusters	0.175	0.107	-0.016	0.164	0.155	0.342	-0.081	0.111	0.051	0.098	-0.601	0.114	0.113	1	0.816	0.376	-0.032	0.357	0.185	0.029
Fragmentation	-0.228	-0.296	-0.282	-0.144	-0.255	-0.124	-0.406	-0.272	-0.293	-0.258	-0.715	0.156	-0.278	0.816	1	0.383	0.101	-0.081	-0.07	-0.046
Compactness	-0.13	-0.327	-0.311	0.035	-0.22	0.039	-0.551	-0.431	-0.457	-0.376	-0.427	0.174	-0.153	0.376	0.383	1	0.14	0.11	-0.01	-0.46
Overlap	0.087	-0.345	-0.565	-0.064	-0.2	-0.305	-0.292	-0.337	-0.319	-0.264	-0.098	0.559	-0.342	-0.032	0.101	0.14	1	0.088	0.065	-0.22
Area synaptic contact	0.905	0.73	0.311	0.587	0.796	0.905	0.566	0.731	0.632	0.671	0.055	0.186	0.611	0.357	-0.081	0.11	0.088	1	0.585	0.14
Axon diameter	0.576	0.433	0.198	0.303	0.471	0.528	0.376	0.467	0.429	0.449	0.057	0.11	0.349	0.185	-0.07	-0.01	0.065	0.585	1	0.161
Fibre diameter	0.221	0.387	0.293	0.066	0.311	0.235	0.42	0.457	0.474	0.429	0.017	-0.051	0.205	0.029	-0.046	-0.46	-0.22	0.14	0.161	1

Heat map of r values:	1.000	.500	.000	-.500	-1.000

Supplementary Figure 5 | Correlations between morphological variables. Correlation matrix obtained from the principal components analysis (PCA), showing Pearson correlation (r) values assessed over the complete dataset of 2,160 NMJs examined. 'Heat map' of r values is shown below the matrix.

	Component	
	1	2
NT-area	.870	-.203
NT-perimeter	.978	-.016
NumTerminalBranches	.740	.250
NumBranchPoints	.695	-.097
TotalLengthBranches	.965	-.096
AChR-area	.867	-.325
AChR-perimeter	.903	.230
EP-diameter	.882	-.061
EP-area	.944	-.044
EP-perimeter	.906	.020
NumberAChR-clusters	.367	.870

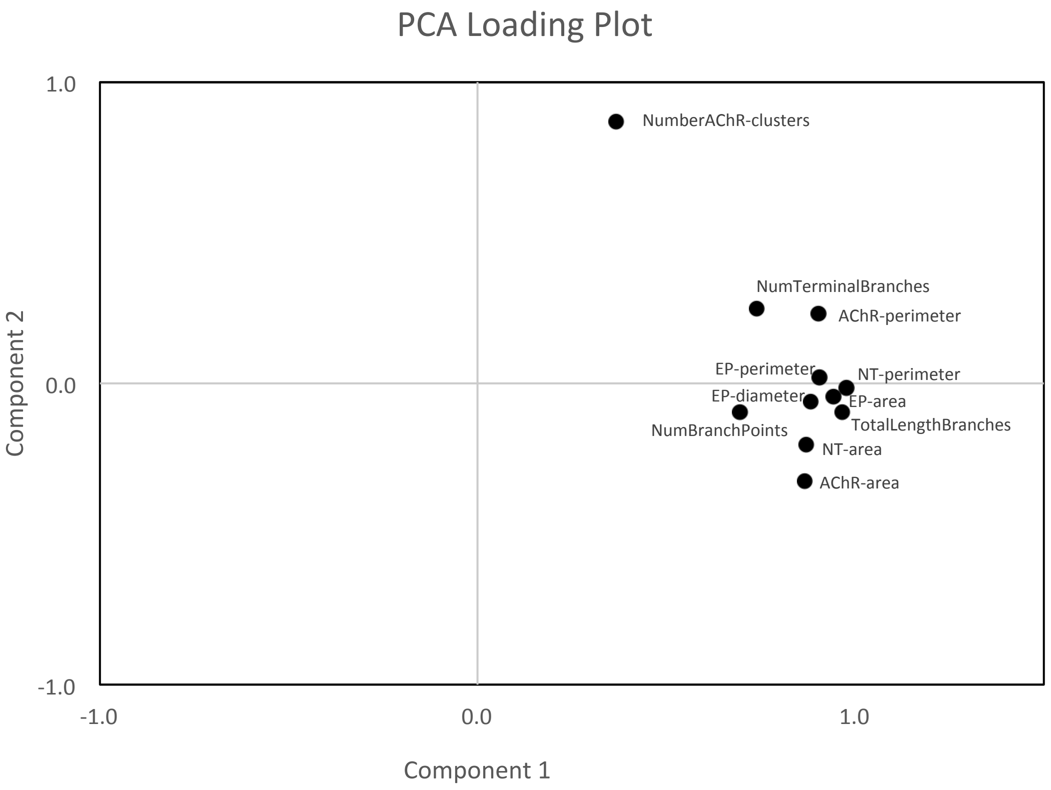

Supplementary Figure 6 | PCA loading plot. Relationship between the morphological variables and the principal components found by 2D PCA. The unrotated loading plot is shown; analysis performed using SPSS.

		AChR area: L vs R		Fragmentation: L vs R	
Muscle	Animal	raw *p*	Significance after Bonferroni correction	raw *p*	Significance after Bonferroni correction
IS	1	0.003	n.s	0.011	n.s
IS	2	0.220	n.s	0.168	n.s
IS	3	0.601	n.s	0.902	n.s
LAL-R	1	0.059	n.s	0.316	n.s
LAL-R	2	0.446	n.s	0.215	n.s
LAL-R	3	0.411	n.s	0.736	n.s
LAL-C	1	0.157	n.s	0.745	n.s
LAL-C	2	0.126	n.s	0.409	n.s
LAL-C	3	0.461	n.s	0.815	n.s
TRI	4	0.944	n.s	0.619	n.s
TRI	5	0.176	n.s	0.127	n.s
TRI	6	0.222	n.s	0.884	n.s
FL2	4	0.001	sig.diff	0.664	n.s
FL2	5	0.549	n.s	0.798	n.s
FL2	6	0.429	n.s	0.973	n.s
FL4	4	0.077	n.s	0.977	n.s
FL4	5	0.026	n.s	0.015	n.s
FL4	6	0.079	n.s	0.165	n.s
QUAD	4	0.690	n.s	0.799	n.s
QUAD	5	0.846	n.s	0.047	n.s
QUAD	6	0.140	n.s	0.510	n.s
HL1	1	0.062	n.s	0.526	n.s
HL1	2	0.666	n.s	0.406	n.s
HL1	3	0.023	n.s	0.934	n.s
HL4	1	0.003	n.s	0.137	n.s
HL4	2	0.033	n.s	0.689	n.s
HL4	3	0.250	n.s	0.202	n.s

Supplementary Figure 7 │ Left/right comparisons of morphological variables within individual animals. Results of statistical tests comparing values of AChR area and fragmentation in the set of 40 NMJs on the left hand side of each animal with the set of 40 NMJs on the right hand side of each animal. For AChR area, an unpaired two-tailed t-test assuming equal variance was used. For fragmentation, the Mann Whitney test was used, as the values are restricted to (0,1). Individual comparisons were regarded as statistically significant using a Bonferroni corrected α value = 0.05/27.
